# SensorDB: a virtual laboratory for the integration, visualization and analysis of varied biological sensor data

**DOI:** 10.1186/s13007-015-0097-z

**Published:** 2015-12-08

**Authors:** Ali Salehi, Jose Jimenez-Berni, David M. Deery, Doug Palmer, Edward Holland, Pablo Rozas-Larraondo, Scott C. Chapman, Dimitrios Georgakopoulos, Robert T. Furbank

**Affiliations:** CSIRO Agriculture, Clunies Ross St, Canberra, 2601 Australia; CSIRO Data61, Clunies Ross St, Canberra, 2601 Australia; CSIRO Agriculture, 306 Carmody Road, Brisbane, 4067 Australia; School of Computer Science and Information Technology, RMIT University, 124 La Trobe Street, Melbourne, 3000 Australia; Centre of Excellence for Translational Photosynthesis, Australian National University, Canberra, 0200 Australia

**Keywords:** Phenomics, High frequency data, Big data, NoSQL, Real-time statistics

## Abstract

**Background:**

To our knowledge, there is no software or database solution that supports large volumes of biological time series sensor data efficiently and enables data visualization and analysis in real time. Existing solutions for managing data typically use unstructured file systems or relational databases. These systems are not designed to provide instantaneous response to user queries. Furthermore, they do not support rapid data analysis and visualization to enable interactive experiments. In large scale experiments, this behaviour slows research discovery, discourages the widespread sharing and reuse of data that could otherwise inform critical decisions in a timely manner and encourage effective collaboration between groups.

**Results:**

In this paper we present SensorDB, a web based virtual laboratory that can manage large volumes of biological time series sensor data while supporting rapid data queries and real-time user interaction. SensorDB is sensor agnostic and uses web-based, state-of-the-art cloud and storage technologies to efficiently gather, analyse and visualize data.

**Conclusions:**

Collaboration and data sharing between different agencies and groups is thereby facilitated. SensorDB is available online at http://sensordb.csiro.au.

## Background

Field based agricultural, forestry and ecology research studies are often undertaken in remote locations and require the collation of varied data types including: time series data from wireless sensor networks; spatial data from imaging devices; human observations scored and recorded on paper or on a portable tablet device; destructive samples and harvests taken from the field and analysed in a laboratory. Such data is typically collated in unstructured repositories on an individual researcher’s computer or on a centrally managed networked file system. Unstructured repositories enable support for varied data types, ease of data modification and analysis in an environment familiar to the user. Hence, these approaches, commonly spreadsheets, are usually preferred over specialized data stores.

However, the use of unstructured, private data repositories does not encourage data sharing and can inhibit effective collaboration. These repositories are usually not scalable beyond a small project or group. As a project becomes larger, the data volume and complexity increases and the task of tracking the location, status and origin becomes difficult and onerous. The potential for data loss, incomplete data recovery and poor reuse of the data thereby increases. Such unstructured data repositories typically do not support data analysis and visualisation for rapid, initial checks of data integrity and are ill suited to large time series data.

SensorDB is a web-based virtual laboratory tool designed to overcome these problems and has been developed through detailed consultation with cross disciplinary researchers working in field based agricultural, forestry and ecology studies. It is designed to receive data from real-time data streams, data upload and download forms and from any geographical location with Internet access. SensorDB is sensor agnostic and supports rapid visualisation of a range of data types. The visualisation framework is designed to be flexible enough to accommodate different types of domain-specific and generic visualisations in a single software solution. As a cloud-based system with a web front-end, it enables data sharing and enhances interaction between different agencies located in multiple geographic locations. Collaborations are thereby facilitated and the turnaround time reduced between data acquisition and data dependent decisions for future experiments and activities.

### Related work

Historically, relational database systems (e.g. http://www.mysql.com) were used to store data streams for future retrieval by users. However, the performance of these systems can be compromised with large time series data and recently, alternative technologies have emerged to address this performance shortcoming. Such technologies are often referred to as *NoSQL databases* to indicate the departure from the SQL programming language traditionally used in the relational database model. An example of NoSQL database technology used in SensorDB is MongoDB (http://www.mongodb.org/), a so-called *document-oriented database*.

The recent technology advances in NoSQL databases and HTML5 interactive data visualization techniques allowed us to build SensorDB. Recently, cloud services have emerged that could potentially support some use cases (e.g. Xively - formerly Cosm and Pachube https://xively.com, and TempoDB https://tempo-db.com), however their direct utility for field based research has yet to be determined.

There has been extensive research on providing efficient data management solutions for sensory data. [1] presented a sensor data collection and visualisation framework comprising of Global Sensor Networks (GSN) middleware and Microsoft SensorMap. SensorMap visualised live sensor data captured by remote sensors on a map oriented interface. In SensorMap, users could find sensors based on their physical location. In comparison with SensorMap, SensorDB’s main focus is providing a virtual laboratory platform where the bulk of the data visualisation and analysis can be performed online. SensorMap was expanded by [2] with the addition of a highly efficient metadata management solution, called *Sensor Metadata Repository*, which provides an advanced metadata search to efficiently discover sensor sources. Using this feature, end-users can identify sensors and deployments based on metadata fields attached to the sensors. While [2] focus on metadata data management, our focus with SensorDB is on real-time statistics. However, the metadata management solution proposed by [2] is complementary to SensorDB and could provide SensorDB with an efficient metadata management and querying solution.

The objective of this paper is to present SensorDB and describe its utility as a web-based virtual laboratory that can manage large volumes of biological time series sensor data while supporting rapid data queries and real-time user interaction.

## Implementation

### SensorDB data model

**Fig. 1:**
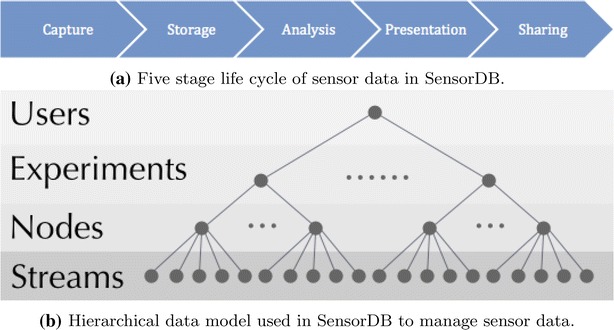
SensorDB data model and data life cycle

The sensor data life cycle in SensorDB consists of five stages and they are depicted in Fig. 1a. The capture stage involves using sensor hardware to measure physical phenomena, for example: air temperature; soil moisture and crop canopy temperature. The storage stage highlights the need to have all captured data and information about the data (metadata), to be stored in a safe location. Examples of metadata information include: sensor types; serial numbers; mac address of sensing devices; experimental treatment; crop sowing date; genotype and replicate number. At the analysis stage, users can mine their data using filtering and grouping by metadata. This process usually involves statistical methods combined with domain-specific knowledge and model-based data analysis tools. The presentation stage is about visualizing the analysis of results. Finally, at the share stage, users can share their data analysis and visualizations with other groups. To achieve this data life cycle, SensorDB uses a hierarchical data model to manage sensor data.

The SensorDB data model consists of the four layers depicted in Fig. 1b. In SensorDB, a user is a logical entity (e.g. a project or a research group), which owns a group of experiments. An experiment has only one owner. Each experiment is a group of nodes and each node belongs to a single experiment. A node can also have its location associated with it, such as latitude and longitude values. A node itself is a group of streams and a stream is a series of timestamp and real number pairs with a unit of measurement. Metadata can be attached at every hierarchical layer in Fig. 1b.

**Fig. 2:**
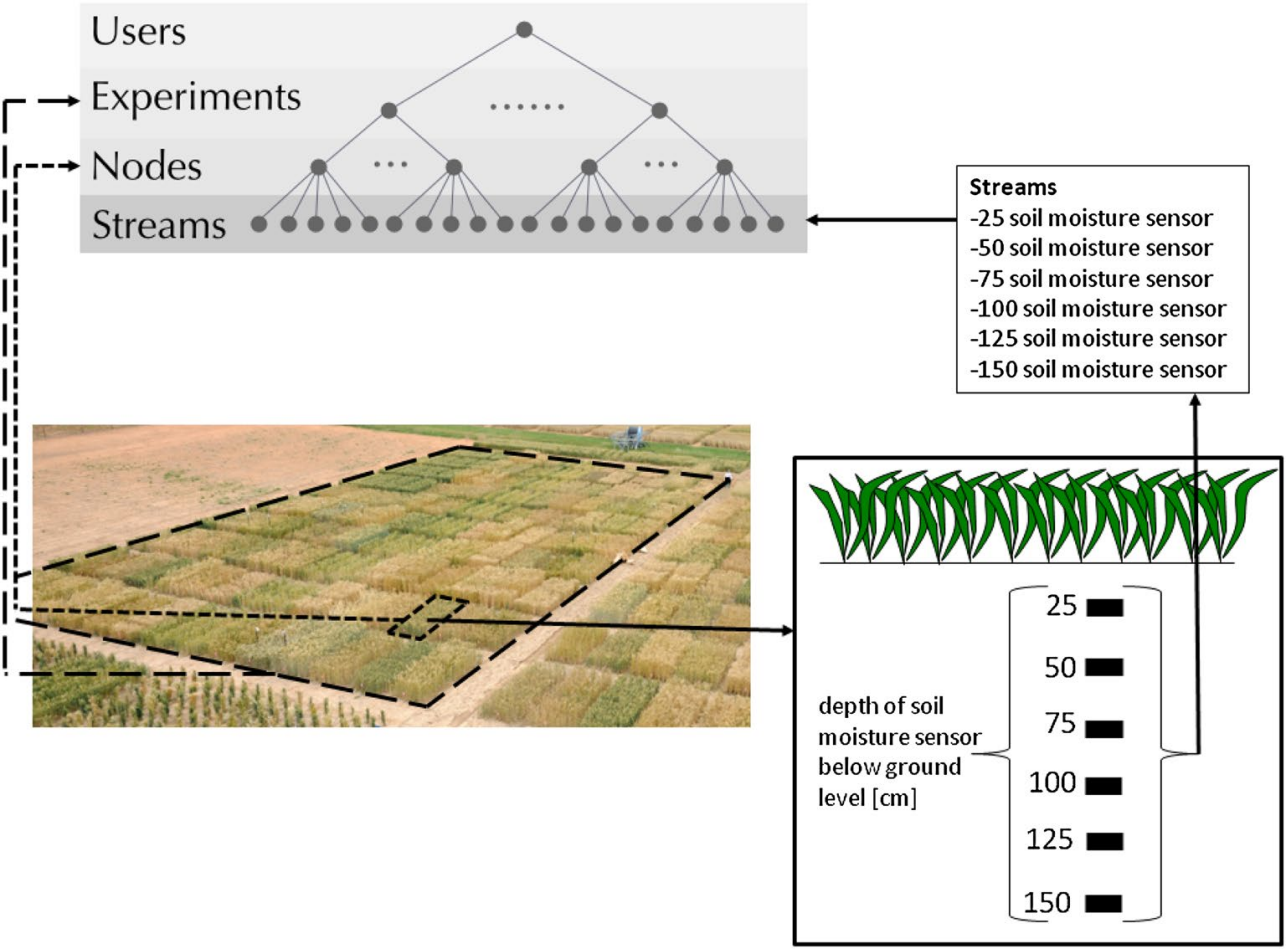
A typical field experiment mapped to the SensorDB data model at the levels: experiment (*long dash*); node (*short dash*) and stream (*solid*)

The mapping of a typical field experiment is illustrated in Fig. 2. In this example, the experiment is an ordered arrangement of ${\sim }$2 m wide by ${\sim }$6 m length experimental units. These experimental units are mapped to the node level in SensorDB. On some of these experimental units, measurements of soil moisture are made at multiple depths. A measurement of soil moisture at a particular depth is mapped to SensorDB at the stream level, associated to a node. The metadata associated to each of the levels in SensorDB is critical for providing contextual information. For the experiment level, metadata could include the following: the year when the experiment was run; the date the experiment was sown; information about the objectives of the experiment and even information about the experimental site like soil type for example. At the node level the most important metadata fields are the treatment (e.g. fertiliser level, genotype, irrigation level) and the relative location of the plot within the experiment (in most cases for this application a row/column notation is used). At the stream level, in this example the depth, the sensor type and the sensor serial number are the most important metadata fields, while sensor information like the date of calibration or settings of the sensor can also be critical.

SensorDB is completely agnostic to the technology used for the data collection. The data model is flexible and supports casting different modalities of data collection from wireless sensor network system to manually taken measurements.

The information is presented to users as a set of time series data. Each time series data corresponds to a physical phenomena measured using sensor networks or manually at an experimental location.

In some cases, sensor information such as battery status, system temperature, data packet loss or radio signal intensity, can become critical for rapidly identifying faulty and potentially faulty sensors and monitoring the system performance over time to maximise data recovery. This information may not be relevant to the scientific investigator, so in our normal operation the information is available as a different diagnostic user or experiment that is separated from the main field experiment. Through separating the system diagnostics from the biological data relevant to the experiment, the end user is thereby presented with a level of abstraction that is focused on deriving meaning from their experiment. This also allows easier maintenance of the automatic data logging systems based on sensor networks where users can get alert email notifications based on a number of criteria like off-nominal values, low battery, missing nodes and time gaps between measurements.

### Software implementation

SensorDB is implemented using the Scala programming language. Scala is an object-functional programming language built on top of the Java virtual machine. Scala provides key constructs that are important for building SensorDB, namely support for lock free multi-threading using actors, to improve performance, and an advanced typing system which can identify a large number of potential system bugs during compile time. Scala also has access to all programming libraries written in the Java language.

**Fig. 3:**
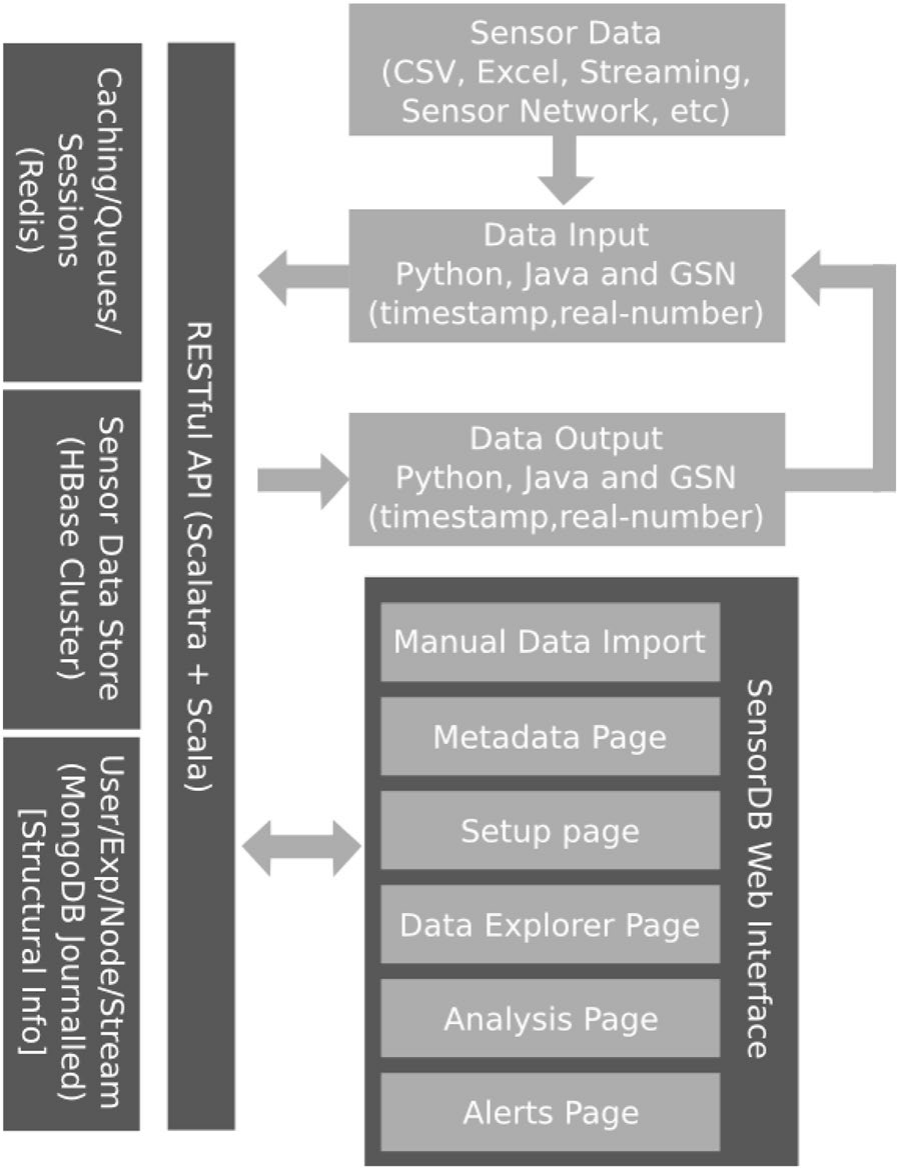
High level architecture of SensorDB

SensorDB is designed to be scalable and to achieve this, it uses a cloud-based architecture combined with asynchronous data processing using persistent queues, stateless worker threads and NoSQL data stores. The high level architecture of SensorDB is depicted in Fig. 3. SensorDB uses NoSQL data stores to manage user data, sensor data, aggregated data and caching of commonly used information. To ensure SensorDB is scalable and can store and serve hundreds of millions of data points, SensorDB internally uses time aggregation windows. There are several fixed time aggregation windows defined in SensorDB; 1-min, 15-min, 1-h, 1-day, 1-month, 1-year and overall. Each time aggregation window maintains critical information about the data points it holds. This information includes the number of data points in a window, minimum and maximum values and the time they occurred, and the mean and standard deviation of the data points inside the window.

The storage layer behind each aggregation window is allocated based on the user and application’s access patterns. Using this approach, one can optimize SensorDB for different application domains. For instance, if a data item at a 1-h aggregation window is accessed more frequently than the 1-min aggregation window, SensorDB can be configured to store and cache data in that aggregation window in a faster storage technology, such as in memory storage or SSD (solid state disk) drive. In the current implementation, the data (and the aggregation window) for 1-min would be stored in the disk level storage layer, such as HBase storage system.

In SensorDB we store three types of information.

1. Structural information including; usernames, passwords, experiment names, node names, node location, stream names, measurement units and metadata information. This information is required to provide context to the sensor data stored in SensorDB.
2. Sensor Data Store containing both raw and aggregated sensor data. This layer is usually backed by a hybrid of storage systems, as described above.
3. Queues, Caching and User Session information. The information includes data that are transient and not critical if lost. This information is calculated using the structural information combined with Sensor data store. Therefore, if it is required, it can be recalculated again, making it suitable for a transient storage layer.

SensorDB is designed so that all the sensor data and information access is done through a RESTful HTTP API using standard HTTP requests and JSON objects. Our choice of using Restful/JSON is based on the ease of use and availability of libraries in different languages such as $Python, Java, R$ to access Restful/JSON based data sources. As such, this architecture allows SensorDB to be a generic system when seen from other platforms. The SensorDB web interface is using the same RESTful/JSON API and illustrates how an application can be built on top of SensorDB’s API. In this model, one can easily swap the existing SensorDB web interface with another solution as long as the new solution adheres to the API defined by SensorDB.

### Data upload

In order to upload sensor data or metadata values to SensorDB, we provide three upload mechanisms:

1. *GSN virtual sensor* GSN [3] is a sensor data processing engine, designed to capture and process real-time data streams. GSN supports more than a dozen classes of sensor hardware and does not require any programming skills to be used, although more intermediate and advanced use cases require knowledge of the Java programming language. Our GSN virtual sensor uses SensorDB’s restful API to push the captured sensor data directly into SensorDB.
2. *Web-based upload for sensor data stored in CSV or MS Excel files* This is the most convenient way of uploading sensor data or metadata, as most of the manual sensor measurements and historical data are normally available in this format. SensorDB’s web interface has a specialized text editor, which parses the CSV and MS Excel file formats. Using this approach, users can simply copy and paste their data files into SensorDB’s web interface and the rest is handled by SensorDB’s web interface.
3. *Script-based upload of streaming or historical data* This is an efficient and scalable way of uploading sensor data or metadata into SensorDB. This approach can be used to upload large quantities of sensor data in batches. It can also be used to capture real-time data streams from sensor hardware for which there is no GSN driver or if the sensor hardware is not directly accessible. Once this approach is combined with task schedules, one can automate the data upload process significantly.

### Real-time statistics on sensor data

A key design requirement of SensorDB was to provide real-time or close to real-time statistical information about the incoming sensor data. As a typical sensor measures thousands to millions of data points during an experiment, from our experience, the patterns and correlations in time resolved data streams are more important than individual data points. This statistical feature is designed to help users to quickly capture the *big picture* of a data stream. SensorDB provides continuous calculation of standard deviation, mean, number of elements, minimum, maximum, last value and last timestamp. This information is calculated at each individual aggregation window and updated with each incoming data point. Moreover, this information at any aggregation window is accessible using SensorDB’s Restful/JSON API.

**Fig. 4:**
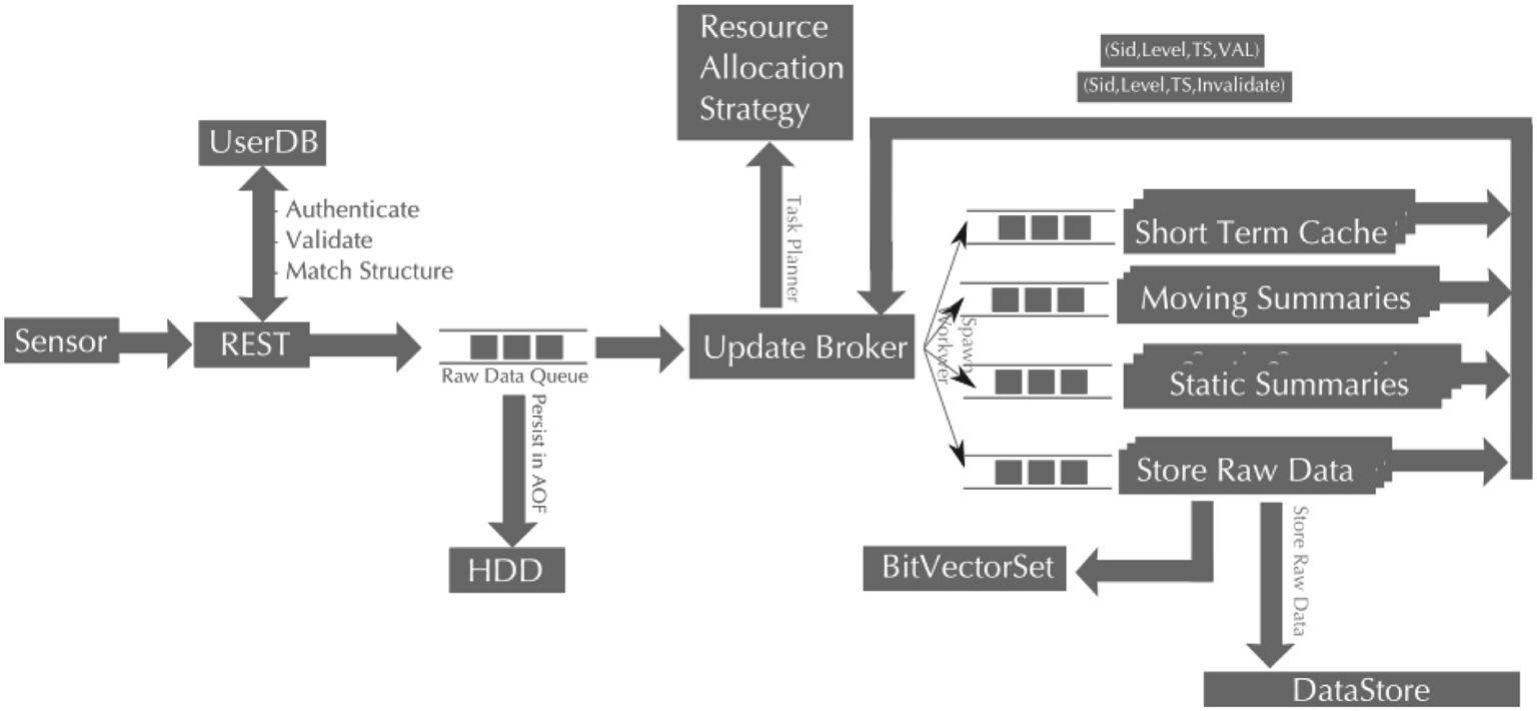
SensorDB data processing model at the stream level

In order to achieve this feature, SensorDB is using a cloud-based elastic data processing model. This approach is depicted in Fig. 4. The unique feature of this architecture is its elasticity, whereby the data processing in SensorDB can be distributed across multiple networked computers. In order to provide high performance throughput, stateless worker threads are used in SensorDB, whereby individual worker threads are not required to access any shared memory space to process their tasks, therefore each calculation is self-contained and performed independently. Using this approach, SensorDB can achieve high levels of parallelism and hence efficiently utilise available computational resources.

With this architecture, each processing node can be deployed on separate computer hardware. If a particular application domain is facing a large burst of sensor data input, one can simply distribute the work across a larger pool of computers to ensure that the system scales well and the system performance is maintained.

### Preliminary analysis, visualization and download features

**Fig. 5:**
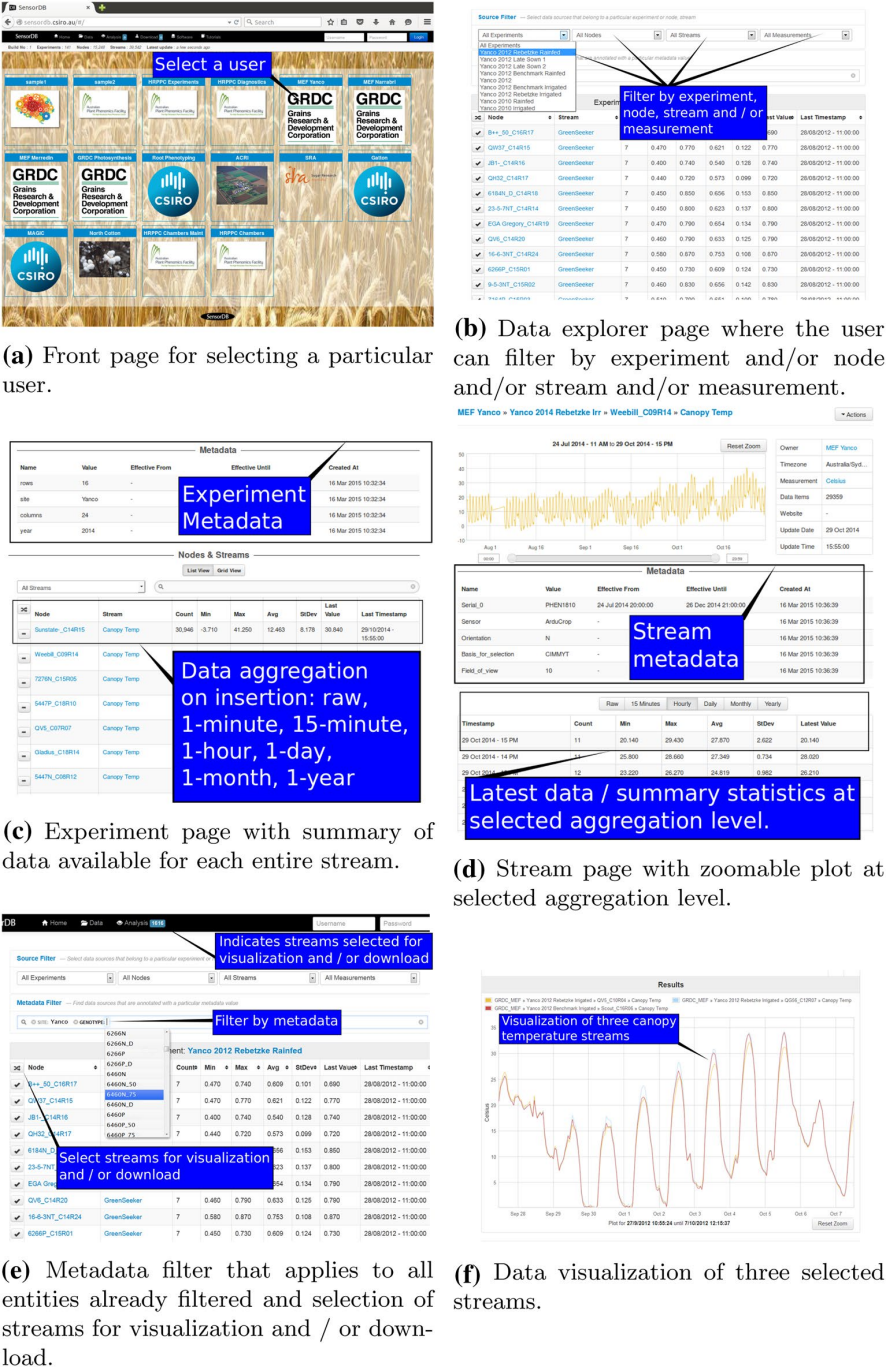
Screenshots of the SensorDB web interface (available at http://sensordb.csiro.au) highlighting key features

Screenshots of the SensorDB web interface are presented in Fig. 5 (available at http://sensordb.csiro.au). The SensorDB web interface is designed to provide the user with an informed view of the data base for preliminary analysis and visualization. A customizable shopping cart approach is used for visualization and download that is user friendly, as it requires no prior software coding skills. A typical SensorDB workflow for preliminary analysis, visualization and download of the data is described as follows. Firstly, the user arrives at the front page and selects their user account (Fig. 5a) which opens a data explorer page containing all of the experiments belonging to that particular user. At this point, it is possible to filter by experiment and/or node and/or stream and/or measurement and/or metadata (Fig. 5b, e).

The user can select an individual experiment and arrive at the experiment page (Fig. 5c) where a summary of the data available for each entire stream can be viewed. Note that for each aggregation level (raw, 1-min, 15-min, 1-h, 1-day, 1-month, 1-year) the following statistics are calculated and updated in real-time: standard deviation; mean; number of elements; minimum; maximum; last value; last time-stamp. At the experiment page the user can also view metadata at the experiment level and filter nodes by stream and/or metadata.

To inspect data from an individual stream, the user can select a stream of interest and arrive at the stream page (Fig. 5d). The stream page comprises the following: the latest summary data and statistics at the selected data aggregation level (raw, 15-min, hourly, daily, monthly or yearly); a zoomable plot at the selected aggregation level; the individual stream metadata. Every stream in SensorDB has a unique stream page.

The user can select multiple streams for visualization and/or download at the data explorer and experiment pages. This is shown in Fig. 5e where selected streams are indicated with a tick symbol and the number of streams selected is indicated at the top of the page. The user then selects “Analysis”, shown at the top of the page in Fig. 5e, and arrives at the analysis page. At this point the user can build a zoomable graph comprising multiple data streams at an aggregation level of interest for data visualization (Fig. 5f). Similarly, a download page is provided whereby the user selects the date range and aggregation level to download selected data streams in CSV file format for customized analysis.

## Results and discussion

### Evaluation of data retrieval latency

The objective of this evaluation was to assess the data retrieval latency of the pre-calculated statistics used in SensorDB and compare it against traditional database solutions where real time calculations are required. For this purpose, a dataset was generated from simulated solar irradiance data with one second frequency using the PySolar Python library (http://pysolar.org/, [4]). Twelve data files were created for one month, two months and sequentially up to twelve months. One month consists of approximately 2.6 million data points, therefore the total amount of data created was 200 million data points. The simulated data was uploaded to SensorDB as well as imported into MySQL and PostgreSQL databases. In the case of SensorDB, one stream was created for each of the simulated files resulting in 12 streams with the different duration of the simulations. In the SQL databases, one table was created for each duration, resulting in 12 different tables.

A SQL query was created for each of the time aggregation levels available in SensorDB, to thereby enable comparison of the data retrieval latency between SensorDB, MySQL and PostgreSQL databases. The SQL queries were implemented using a GROUP BY clause with the difference in time between the data timestamp and a fixed date. Given the differences in the SQL syntax for the different engines, each query was slightly different while using the same logic (Table 1). The same statistics that are available in SensorDB for the aggregated data where calculated in the SQL queries: average; min; max; count; standard deviation.

**Table 1:**
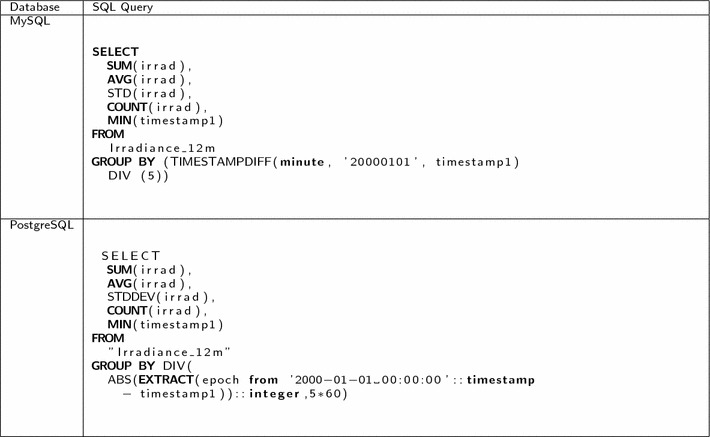
SQL queries for calculating the different levels of time aggregation

Example of SQL queries used for calculating time aggregation in MySQL and PostgreSQL. This example shows the aggregation of 5 minutes over the 12 months table Irradiance_12m. Different levels of aggregation can be obtained by changing the value of the minutes used in the DIV operation

**Fig. 6:**
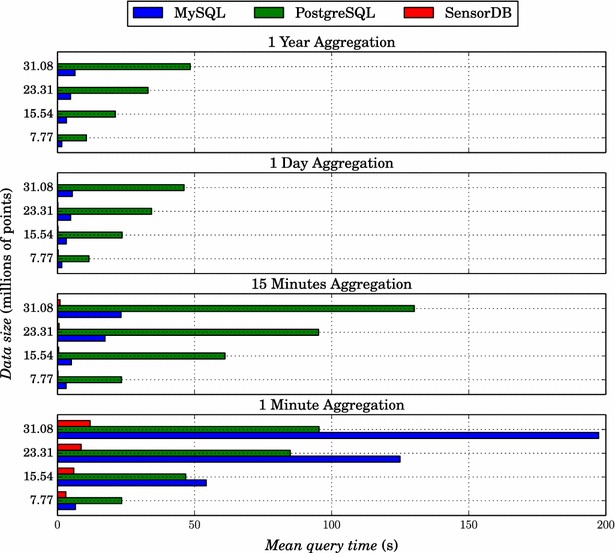
Benchmark of SensorDB, MySQL and PostgreSQL. Query times for different data sizes and aggregation levels using; **a** MySQL, **b** PostgreSQL and **c** SensorDB. Note that for the 1-year and 1-day aggregation levels, the query time for SensorDB was less than one second

In order to time the results consistently, the same server was used for the three tests (Dell T7600, 128 Gb RAM, 2x Intel Xeon CPUs with a total of 16 cores). For each time aggregation level and data size, the mean time for three repetitions of the query was calculated. The results (Fig. 6) show that the query times for SensorDB were faster, by one to two orders of magnitude, than the other databases for each data size at all the aggregation levels. Moreover, for a given aggregation level, the query time increased linearly with data size for MySQL and PostgreSQL. In contrast for SensorDB, aside from the 1-min aggregation level, the query time was approximately constant and close to one second for all the aggregation levels and data sizes. In the case of PostgreSQL it was approximately 20 times slower than MySQL for all the aggregation levels except for the 1-min level. At the 1-min aggregation level, the performance of MySQL degraded in comparison to PostgreSQL.

### Evaluation of SensorDB scalability

SensorDB has been designed with scalability in mind. A benchmarker was developed to test the performance of SensorDB when accessed by several clients simultaneously. The benchmarker acted as a client to the SensorDB RESTful interface, via the Java API. A suite of benchmarks consisted of collection of configurations for read and write testing. Individual configurations contained specifications for the number of experiments, nodes, streams, data size and client threads, along with a number of other parameters, that a test run would use.

The benchmarker would then use the configuration to generate a series of requests—simultaneous requests in the case of multiple threads—for the server to process. A request consists of the following: the API being invoked with some parameters; converting the parameters into a JSON document suitable for transmission to the server (“marshalling”); making a HTTP request to the SensorDB server; waiting for the server to process the request; receiving the response from the server in the form of a JSON document and converting the response document from JSON back into a Java object (“unmarshalling”).

The time spent making the request to the server and processing within the API—marshalling and unmarshalling—were both recorded and statistics collected. In all cases, the time spent by the API marshalling and unmarshalling requests and data was less than 2 % of the total request time and has been ignored. The *request time*, therefore, is the time from the start to the end of communication with the server. In cases where the majority of time is spent waiting for the server to finish processing and return a result, the term *response time* is occasionally used to emphasise that the client is waiting on the server—and thus free to perform other processing in other threads. Request time and response time both refer to the same measurement.

The *total run time* is the time spent running an entire benchmark, from the time the first client thread starts to the completion of all client threads. In the case of multiple, parallel threads, the total run time simply refers to the elapsed (clock) time, not the sum of the run times of each thread.

The benchmark server was the Dell T7600 described in the previous section. The client machine was a Dell M4800 workstation (16 Gb RAM, 1x Intel i7 2.80 GHz CPUs with a total of 4 cores). The client and server were connected by the 1GBs CSIRO internal network.

#### Write scalability

**Fig. 7:**
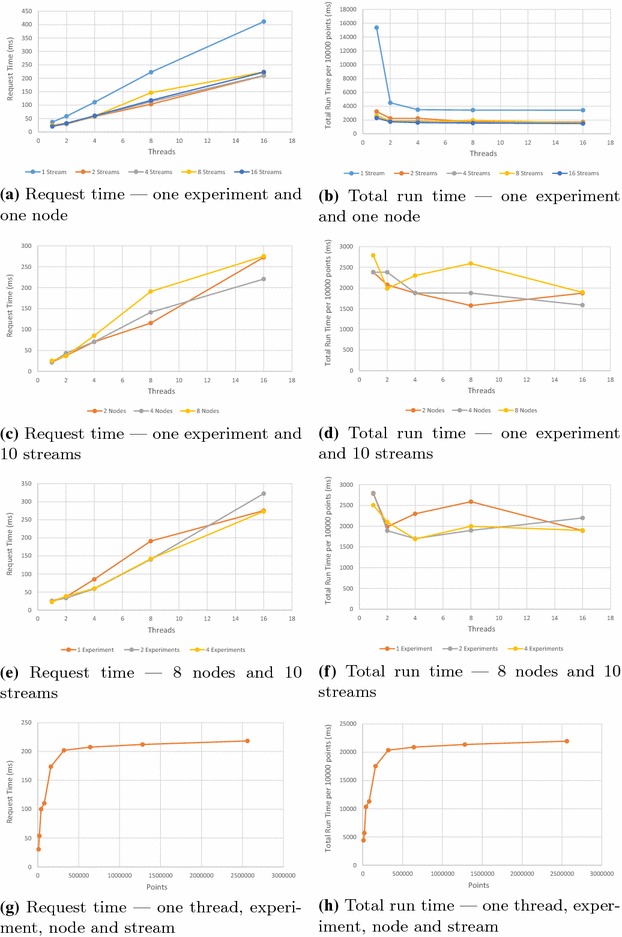
Write benchmarks for varying numbers of client threads, experiments, nodes, streams and points inserted

In the write tests, each stream had a fixed number of data points written in blocks of 100 points. Generally, each stream received 10,000 points. Since the benchmarks vary in the number of streams, a normalised total run time per 10,000 points is shown. The results of the write benchmarking tests are shown in Fig. 7.

The results for varying stream numbers for different numbers of client threads are shown in Fig. 7a, b. With the exception of a single stream, similar request times and run times occur across increasing numbers of streams. Increasing client numbers increases the request time, due to contention at the server; the data store has a single write lock and requests need to be queued until processed. A single stream is consistently slower than multiple streams, since the server may need to lock overlapping data segments. The unusually slow throughput for a single thread is caused by the thread waiting for a response before proceeding with the next request; with two or more threads, a new request is already queued for the server to process when the response to a request has been sent. Otherwise, total throughput remains essentially constant.

The results for varying node and experiment numbers are shown in Fig. 7c–f. Altering the number of nodes or experiments shows no significant variation in performance.

The result for inserting growing numbers of points into a stream are shown in Fig. 7g, h. In this test, large numbers of points were inserted as quickly as the server permitted. SensorDB performs well when inserting relatively small numbers of points. After about 160,000 points, the server becomes saturated and throughput becomes constant.

#### Read scalability

**Fig. 8:**
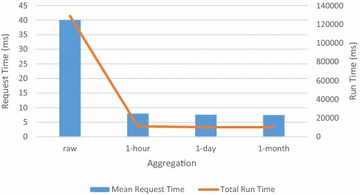
Read benchmark for varying aggregation types—one thread

**Fig. 9:**
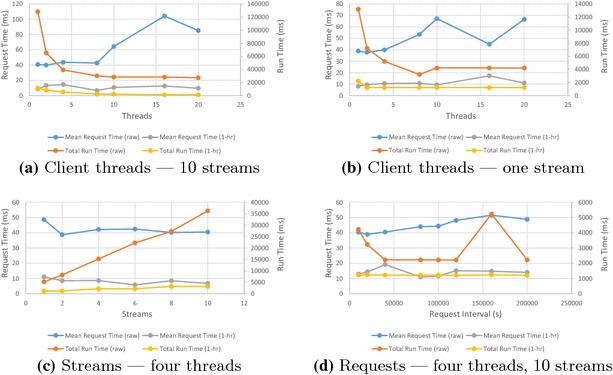
Read benchmarks for varying number of client threads, streams and request sizes

The read tests started by populating a SensorDB experiment with 10 streams across 5 nodes, with each stream containing 1,000,000 values at one second intervals. The results of the read benchmarking tests are shown in Figs. 8 and 9.

SensorDB allows read queries across several aggregation types. Fig. 8 shows the request time and total run time for raw data, as well as data aggregated over hourly, daily and monthly intervals. There is little difference between the different aggregation intervals. Since there are 3600 raw points for every hourly summary, the server takes longer to assemble the result. The subsequent benchmarks show results for both raw data and hourly aggregated data.

Figure 9a shows the effect of multiple clients simultaneously requesting data spread across 10 streams. Figure 9b shows the effect of multiple clients simultaneously requesting data from a single stream. The total run time drops as the number of threads increases, showing that the server is handling simultaneous requests with a high degree of parallelism. For raw data, request time rises after eight threads. Since there seems to be no major overhead in unmarshalling the received data, total run time remains constant beyond this point and the client machine became CPU-limited beyond 8 threads, this result appears to be largely the result of client machine limitations.

Figure 9c shows the effect of multiple simultaneous reads over varying numbers of streams. With the exception of a small amount of contention for a single stream, there is little difference between results.

Figure 9d shows the effect of increasing request size. Requests are made over a time interval; the x-axis shows the interval size in seconds. There is a slight rise in the time taken to assemble increasing amounts of raw data. Otherwise, SensorDB is insensitive to request size.

#### Large numbers of streams

**Fig. 10:**
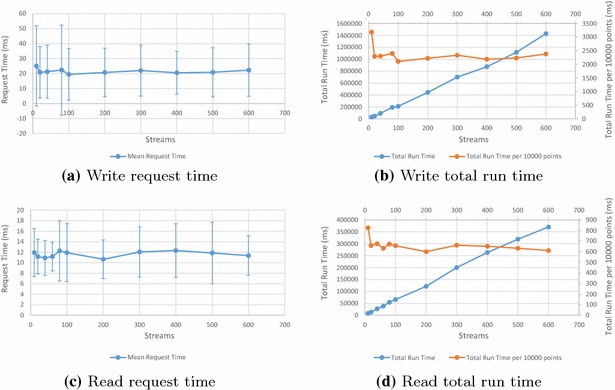
Write and Read Benchmarks for varying numbers of streams. Write Benchmark—one thread and experiment, 10 nodes. Read Benchmark—one experiment, 10 nodes, 4 threads

Two benchmark suites were run to investigate the effect of large numbers of streams on SensorDB. In both cases, a varying number of streams were spread across a single experiment and 10 nodes, with each stream containing 10,000 values. The results for writing and reading varying numbers of streams is shown in Fig. 10. The request time plots show error bars for a single standard deviation (Fig. 10a, c); as can be seen, response time is quite variable. Altering stream numbers shows little effect on performance.

### Case studies

A design objective of SensorDB is to empower its users by providing common tools combined with a flexible yet powerful API. To achieve this goal, SensorDB’s team maintains an expanding list of official SensorDB API implementation in commonly used languages. As of today, APIs exist for Python and Java languages. As a demonstration of the utility of the tool and user empowerment, we present case study applications built by SensorDB users independently and in parallel of SensorDB’s core development agenda. These case studies are available at http://www.sensordb.csiro.au.

#### Case study 1: Diagnostic user (SensorDB mapped with respect to the sensor hardware)

This application utilised SensorDB as a diagnostic tool for a wireless sensor network deployed on a field experiment. The deployment comprised of 170 thermopile based infra-red temperature sensors to measure crop canopy temperature [5]. Each sensor reported a measurement of battery voltage, sensor body temperature and object temperature every 15-min. Each day, the data was uploaded to SensorDB and an email error report sent to the staff responsible for maintaining the deployment. The email error report contained the following information: gap error (a list of sensors where the time between successive readings exceeded a predefined time); min and max error (a list of sensors where the minimum and maximum object temperature was below or above a predefined temperature) and missing sensor error (a list of sensors that failed to report data during the previous day). This approach minimised data loss by identifying faulty sensors as early as possible. Whilst this approach enables users to indentify faulty sensors, there is no feature implemented within SensorDB to tag faulty values and thereby exclude such values from further evaluation. However, it is possible for the user to programmatically extract data from SensorDB, “clean” the data using an appropriate algorithm to remove spurious values and reingest the data as a new stream.

#### Case study 2: Biological user (SensorDB mapped with respect to the biological experiment)

For the same field experiment described above in case study 1, the object temperature data (i.e. the biological data of interest) was mapped to the SensorDB data model with respect to the experiment (in Fig. 2). The metadata for each node was uploaded to provide contextual information for the stream data. In this case, each node is an individual experimental unit and the stream is the time series of object temperature. Note that each node can have multiple streams. Mapping the experimental field plan and sensor data to the SensorDB data model with respect to the biological experiment in this fashion, permits the researcher to think in terms of the experiment in a more natural way and immediately provides contextual information to the sensor data streams. Visualisation and comparison of treatments and experimental units was then straight forward.

#### Case study 3: Visualisation of data from multiple sensor types

In this case study we used the Restful/JSON API through a Python toolkit to retrieve data from different sensor types. The RESTful/JSON API is a powerful feature of SensorDB that we utilise through a REST client developed in Python for script based transactions with SensorDB. The Python REST client enables users to develop their own scripts for SensorDB data upload and download, without directly interacting with the core development team. The Python toolkit currently supports data upload for the following sensors: Vaisala WXT520 weather station http://www.vaisala.com/; Hussat soil moisture sensor http://www.hussat.com.au/; the GreenSeeker normalised difference vegetative index hand held sensor http://www.ntechindustries.com/RT100-handheld.html; an infra-red temperature sensor developed in-house and Licor LI-191R line quantum sensor http://www.licor.com/env/products/light/quantum_line.html.

## Conclusions

The recent advent of affordable sensor hardware has meant that obtaining time series data of physical phenomena is now straight forward and routine. Hence, sensor data is undisputedly one of the growing sources of information for many companies and government agencies worldwide. The challenge has shifted from development of sensing hardware for data collection, to development of data handling systems to enable rapid interpretation and synthesis of the data by multiple users. This new challenge is evident in the agricultural research area of field based phenomics, where crop improvement is sought through a greater understanding of gene function and environmental response [6], as a recent review identified that effectively managing the data streams is a major research priority [7]. Such phenotyping research studies are often undertaken in remote locations representative of the target environment [8] and increasingly require multidisciplinary collaboration among researchers [9] to collect, process and analyse the varied data types. Furthermore, continuous measurements of crop canopy temperature in irrigated cotton show promise as a decision support tool for commercial producers [10]. The sheer magnitude of the number of observations generated in the aforementioned research and industrial applications can overwhelm traditional relational databases and unstructured data management systems. In contrast, SensorDB offers a solution that provides the user with real-time statistics at various aggregation windows through a cloud service.

The focus of SensorDB on statistical information allows our users to quickly explore their data, discovering the trends and irregularities. SensorDB supports powerful stream data processing tools, including indexing with user-defined metadata, that enable users to rapidly aggregate, select, group and filter their data without programming. The virtual laboratory environment of SensorDB empowers the end user to focus on the meaning of the data and maximise value extraction. It also allows sharing of big data collected from sensors, as well as analytical results, increasing productivity and know-how via collaboration through sharing any individual’s data and analysis algorithms. Via the elastic cloud solution used here, SensorDB scales as required by the application without the necessity to redesign. As a ready to scale product, SensorDB guarantees instantaneous response to user demands for data and big data analysis. This means outstanding throughput is preserved during data access regardless of the volume of data stored in the system. The *do it yourself* modular nature of SensorDB allows it to be easily adopted to suit varied applications without having to directly interact with the core development team. Using this approach, we are empowering a community of users.

In conclusion, SensorDB is a novel web based virtual laboratory tool for managing large volumes of biological time series sensor data while also supporting rapid data queries and real-time user interaction. Users are empowered through a Restful HTTP API that supports script based user interaction for customised visualisation, analysis and data IO. Collaboration and data sharing between different agencies and groups is facilitated through the use of a web based tool with a structured data model.

## Availability and requirements

Project name: SensorDB. Project home page: http://sensordb.csiro.au. Operating system(s): Platform independent. Programming language: Scala, Python, Java. Other requirements: http://sensordb.csiro.au is compatible with the following web browsers: Chrome; Firefox; Internet Explorer 10; Internet Explorer 11. Any restrictions to use by non-academics: licence required.
